# Feasibility of Methylated *CLIP4* in Stool for Early Detection of Colorectal Cancer: A Training Study in Chinese Population

**DOI:** 10.3389/fonc.2021.647066

**Published:** 2021-04-22

**Authors:** Yang Cao, Guodong Zhao, Yaping Cao, Zhiliang Chen, Xiaoyu Liu, Mufa Yuan, Jun Yang, Xiaomei Wang, Yong Ma, Zhaocheng Liu, Shangmin Xiong, Minxue Zheng, Sujuan Fei

**Affiliations:** ^1^ Department of Gastroenterology, Affiliated Hospital of Xuzhou Medical University, Xuzhou, China; ^2^ Institute of Digestive Diseases, Xuzhou Medical University, Xuzhou, China; ^3^ Zhejiang University Kunshan Biotechnology Laboratory, Zhejiang University Kunshan Innovation Institute, Kunshan, China; ^4^ State Key Laboratory of Bioelectronics, School of Biological Science and Medical Engineering, Southeast University, Nanjing, China; ^5^ Department of R&D, Suzhou VersaBio Technologies Co. Ltd., Kunshan, China; ^6^ Suzhou Institute of Biomedical Engineering and Technology, Chinese Academy of Sciences, Suzhou, China

**Keywords:** colorectal cancer, early detection, stool, *CLIP4*, DNA methylation

## Abstract

**Background:**

Early detection of colorectal cancer (CRC) and precancerous lesion is vitally important for mitigating CRC morbidity and mortality. Aberrant DNA methylations in certain promoter regions have been identified to be closely associated with CRC development and progression, suggesting their potential as diagnostic biomarkers for early detection. In this study, we evaluated the performance of methylated *CLIP4* in stool specimens as a potential biomarker for CRC detection.

**Methods:**

A total of 321 subjects out of 365 enrolled participants were included in the final analysis, including 154 CRC patients, 23 advanced adenoma (AA) patients, 49 small polyp (SP) patients, and 95 healthy controls. *CLIP4* methylation level was examined by qPCR with bisulfite converted DNA purified from approximately 5 g stool specimen.

**Results:**

Methylated *CLIP4* test showed high sensitivities of 78.3% (95% CI: 55.8%–91.7%) and 90.3% (95% CI: 84.2%–94.3%) for detecting AA and CRC, respectively, with a specificity of 88.4% (95% CI: 79.8%–93.8%). *CLIP4* methylation level discriminated AA and CRC patients from control subjects with area under the curve values of 0.892 (95% CI: 0.795–0.988) and 0.961 (95% CI: 0.938–0.983). Further analysis indicated no significant difference in sensitivities among different ages, genders, stages, locations, sides, tumor sizes and differentiation statuses.

**Conclusions:**

Methylated *CLIP4* showed a strong potential as a noninvasive biomarker for early CRC detection.

## Background

Colorectal cancer (CRC) remains the third most commonly diagnosed cancer types, and the second most common cause of cancer-related deaths worldwide in 2020 (1). In China, new CRC cases and death in 2020 were 555,477 and 286,162, accounting for approximately 29% of the global disease burden. Rankings of CRC rose from the fifth most common cancer before 2015 to the second in both sexes. Incidence rate of CRC in China exhibited a substantial upward trend in the past decades, and age-standardized mortality rate also endured an upward swing (2). Meanwhile, significant and sustained declines in both incidence and mortality for adults over 50 years old have occurred in the United States, owing to increased awareness of screening, especially by colonoscopy (3, 4).

Multiple CRC screening methods have been developed over the years, and each has its own advantages and disadvantages. For adults over 50 years old in the US, routine fecal occult blood test (FOBT) or fecal immunochemical test (FIT) (5), sigmoidoscopy, colonoscopy, computed tomography colonography or stool DNA (sDNA) test are recommended for CRC screening (3, 4). Similar screening methods are recommended for high-risk group over 40 years old and low-to-average-risk group over 50 in China (6). As the gold standard, colonoscopy has higher sensitivity and specificity than stool-based tests, especially for precancerous lesion and early stage CRC. However, the population coverage in China is still insufficient. The compliance rates for colonoscopy and FOBT remained at low levels of 4.01% and 11.01%, respectively, even in Shanghai, one of the most developed Chinese cities in the past decade (7–9). A large screening campaign of 182,927 participants with high-risk for CRC from 16 Chinese provinces only increased the compliance rate for colonoscopy to 14.0%. History of FOBT or colonic polyp, family history of CRC and high level of education were found to be associated with the increased participation (10).

In the meantime, existing stool-based tests providing non-invasive and high-compliance alternatives bear their own drawbacks. FOBT or FIT for fecal hemoglobin detection is affordable, but their performance is unsatisfactory due to low sensitivity in detecting advanced colorectal neoplasia. Cologuard, the first stool-based CRC screening test approved by the US Food and Drug Administration (FDA), demonstrated relatively high sensitivity and specificity (11). However, a high list price of $649 due to its complex operations associated with multiple assays per test makes it difficult to promote among uninsured and/or low- and moderate-income population. Therefore, intensive efforts have been made to develop more accurate and cost-effective screening tests.

DNA methylation is an epigenetic mechanism of gene regulation. Aberrant DNA methylation has been observed in all cancer types including CRC (12). Therefore, it has emerged as a class of important biomarkers with more diagnostic values than mutation markers for early CRC detection (13). A number of methylated genes have been proposed as CRC biomarker candidates in previous studies. Several among them have been incorporated into one-marker or multi-marker commercial tests, such as methylated *SEPT9*, *SDC2*, *SFRP2*, *VIM*, *BMP3*, and *NDRG4*. Another new candidate, *CLIP4*, is a member of CAP-Gly domain containing linker protein (*CLIP*) family involved in plus-end binding of microtubule, and has been implicated in immune response-related biological processes, cell migration and viability in certain cancer metastases (14, 15). Hypermethylation of *CLIP4* in plasma has been shown for cancer types such as CRC and gastric cancer. Further studies in multiplex blood-based methylation tests validated its potential as another promising biomarker for CRC (16–19). However, the performance of methylated *CLIP4* (m*CLIP4*) in stool samples for CRC detection has never been reported. The aim of this study was to evaluate the feasibility of stool m*CLIP4* as a biomarker for early CRC detection.

## Materials and Methods

### Sample Collection

The original plan was to perform stool m*CLIP4* test on 400 participants at the Affiliated Hospital of Xuzhou Medical University, comprising 200 CRC patients, 100 polyp patients and 100 subjects with no evidence of disease (NED). The inclusion criteria consisted of the following: 18 years old or older, no history of CRC, no pregnant woman; and all participants must have undergone complete colonoscopies by trained physicians. Standard operation was followed for all colonoscopy examinations where endoscope reached cecum. Participants with abnormal colonoscopy results should have pathological diagnoses. Pathology analysis was first done independently by two trained pathologists. If both agreed on the same diagnosis, no further evaluation was needed. If their diagnoses did not agree, evaluation by a third pathologist was required for the final determination of diagnosis. All pathologists involved were at or above the level of associated chief pathologist. During stool sample collection, transferring urine into the collection tube was avoided, and no diarrhea sample was collected. All samples were collected before purgative bowel preparation for colonoscopy. Approximately 5 g of solid specimen was collected from whole stool and preserved in 25 mL of preservative buffer (Suzhou VersaBio Technologies Co., Ltd., Kunshan, China) in a 50 mL tube to stabilize human genomic DNA. Stool samples were stored at room temperature for at most 7 days before being transferred to −80°C for long-term preservation and storage.

**Figure 1:**
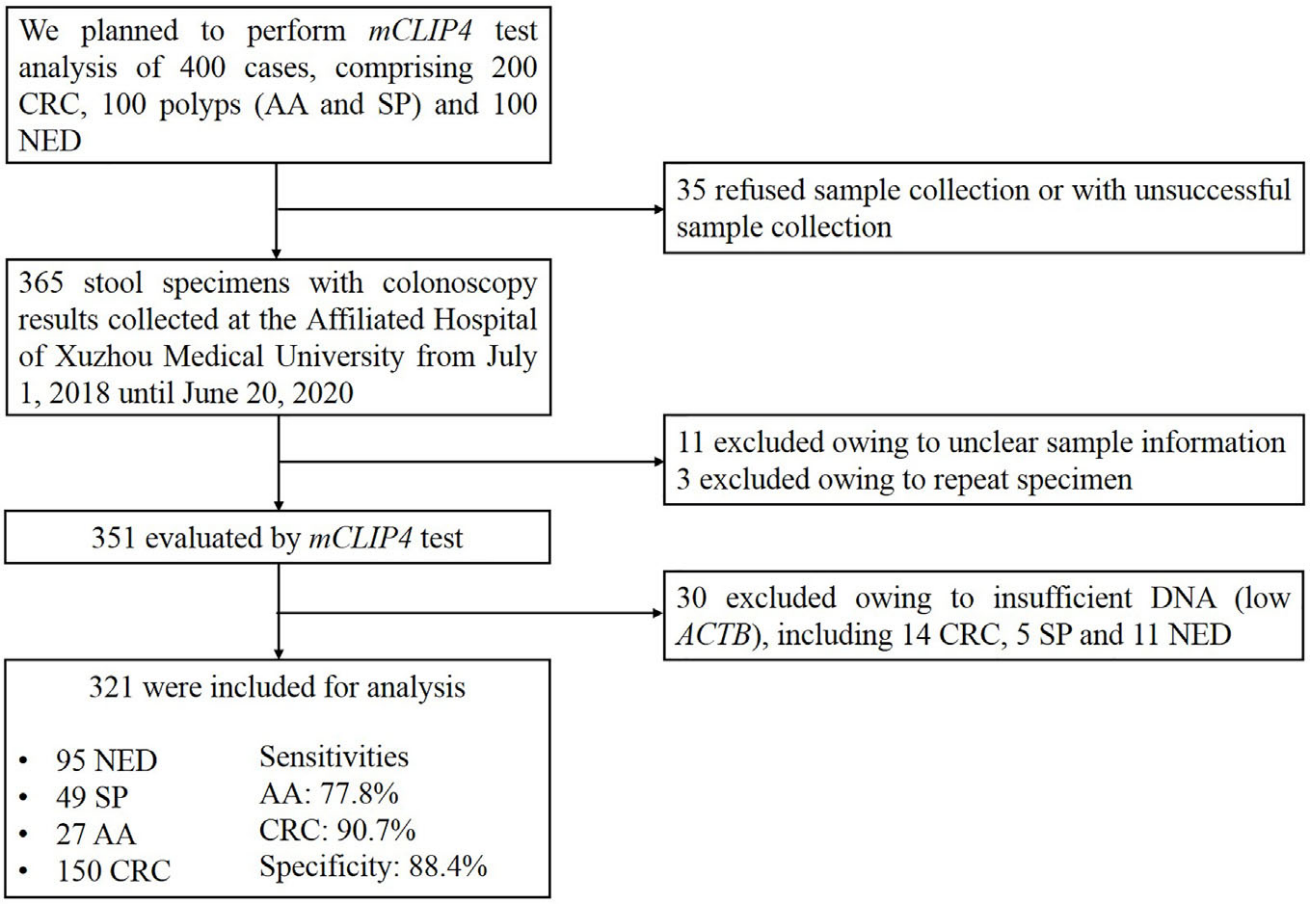
Flow diagram of the study.

Until the submission of this manuscript, 365 stool specimens were collected, among which 11 were excluded due to insufficient sample information and another three were excluded due to repeated sampling. Of the remaining 351 specimens evaluated by m*CLIP4* test, 30 samples were excluded due to insufficient DNA indicated by low *ACTB* levels (see data analysis). As a result, the final analysis included 321 specimens collected from 154 CRC patients, 23 patients with advanced adenomas (AA, an adenoma measuring ≥ 10 mm in size, with high-grade dysplasia, or with ≥ 25% villous features), 49 with small polyps (SP, non-advanced adenoma or hyperplastic polyp) and 95 NED control subjects (**Figure 1**).

Fresh-frozen CRC tissues (n=28) and paired adjacent paracancerous tissues (n=28) were collected at the time of surgery at the Affiliated Hospital of Xuzhou Medical University. The details of age and gender distribution of tissue samples were described in **Supplementary Table 1**. All tissue samples were stored at −80°C until use.

This study was performed according to the principles of the Helsinki Declaration and approved by the Institutional Review Board of the Affiliated Hospital of Xuzhou Medical University (Ethics Committee reference number: XYKY2020-KL156-01). All participants have acknowledged and signed the informed consent.

### DNA Extraction, Bisulfite Treatment and Quantitative Real-Time PCR

Tissue genomic DNA was extracted with a DNeasy Blood & Tissue Kit (Qiagen, Hilden, Germany) according to the manufacturer’s protocol, and purified DNA was eluted into 200 μl Buffer AE. DNA concentration was quantified with an Invitrogen NanoDrop One Microvolume UV-Vis Spectrophotometer (Thermo Fisher Scientific, Waltham, Massachusetts).

Stool specimens were first thawed for approximately 30 min at 15°C to 30°C and homogenized for 1 min on a shaking device. After centrifugation at 10,000 g for 20 min, human genomic DNA was isolated with a stool DNA extraction kit (Suzhou VersaBio Technologies Co., Ltd.) from 150 μl supernatant. Bisulfite conversion of the extracted DNA and purification of the converted DNA were performed with a bisulfite conversion kit (Suzhou VersaBio Technologies Co., Ltd.). Both kits were used according to previously published protocols (20).

Converted and purified DNA was then tested by a duplex qPCR assay. Tissue genomic DNA was tested in a single PCR reaction, and stool DNA was tested in three PCR replicates for m*CLIP4* and an internal control (*ACTB*). The primers and probes used for m*CLIP4* qPCR test were showed in **Supplementary Table 2**. The total reaction volume was 30 μl including 15 μl DNA. qPCR was performed on an ABI 7500 instrument (Applied Biosystems, Foster City, CA, USA) under the following conditions: initial activation at 95°C for 20 min, followed by 50 cycles at 95°C for 10 sec, 60°C for 30 sec and 72°C for 15 sec, and a final cooling to 40°C for 30 sec.

The target sequences of HCT116 and Jurkat genomic DNA were determined by bisulfite Sanger sequencing. Bisulfite-treated DNA was amplified with primers flanking the target region (**Supplementary Table 2**), and the expected size of the PCR product was 263 bp. PCR cycling conditions were as follows: initial activation at 95°C for 15 min, followed by 50 cycles at 95°C for 1 min, 58°C for 45 sec and 72°C for 30 sec, and a final cooling to 4°C for 1 min. PCR products were excised from agarose gels and purified with AxyPrep DNA Gel Extraction Kit (Axygen Biosciences, Hangzhou, China). Purified PCR products were ligated into pUCM-T vector (Sangon Biotech) at 16°C overnight and then transformed into competent *E. coli* cells (Tiangen Biotech, Beijing, China) according to the manufacturers’ instructions. Randomly selected transformants were subsequently sent for Sanger sequencing with the canonical MF-13 primer by Genewiz, Inc. (Suzhou, China). The agarose gel electrophoresis and Sanger sequencing results were shown in **Supplementary Figures 1** and **2**.

### Limit of Detection of m*CLIP4* Assay

Limit of detection (19) of m*CLIP4* test was evaluated with a series of mixtures between fully methylated HCT116 genomic DNA and unmethylated Jurkat genomic DNA at different ratios (0, 6.75, 12.5, 25, 50, 60, and 70 pg fully methylated DNA out of 70 pg total DNA per qPCR reaction). The test was performed in 24 replicates for each mixture. Genomic DNA concentration was measured by an Invitrogen Qubit fluorometer and a Qubit dsDNA HS Assay Kit (Thermo Fisher Scientific, Waltham, Massachusetts).

### Data Analysis

Ct values of *ACTB* and m*CLIP4* were obtained to validate sample processing and to determine whether m*CLIP4* was detected, respectively. The results for stool specimens were considered ‘valid’ if all three replicate reactions for *ACTB* produced amplification signals and the mean Ct value was less than 40.0. To be scored positive by 3/3 algorithm, all three replicate m*CLIP4* PCR reactions of a stool sample must have valid amplification curves and mean Ct value must be less than 39.0. Sensitivity was defined as the positive detection rate of CRC or AA and specificity was defined as 100% minus the positive detection rate of NED.

All statistical analyses were performed with IBM SPSS for Windows Version 22.0. *Pearson* chi-square test for sensitivity comparisons among groups was performance at a significant level of *p* < 0.05. And the differences in methylation levels were analyzed using the Mann–Whitney U test. ΔCt was used to determine the methylation levels of *CLIP4* in tissue samples. It was defined as the difference between the Ct values of the target (m*CLIP4*) and the internal control gene (*ACTB*) to normalize for DNA amounts of tissue samples. Mean Ct values from individuals in CRC, AA and control groups were used to plot the receiver operating characteristic (21) curves and to calculate the area under the curve (22) values. Ct values of reactions returning no amplification signals were set to 50 (the maximal number of PCR cycles) for the analysis (23). Mean Ct values were also used to represent the methylation level of each plasma sample.

## Results

Twenty-eight colorectal cancer and paired adjacent paracancerous tissues were collected, including 16 males of 33 to 78 years old (**Supplementary Table 1**). Stool specimens were evaluated by m*CLIP4* test for 321 subjects (**Table 1**), including 95 control (NED) subjects, 49 SP patients, 23 AA patients, and 154 CRC patients at median ages of 48, 55, 66 and 62.5, respectively. The CRC patients included 4 Stage 0, 26 Stage I, 48 Stage II, 48 Stage III, 10 Stage IV and 18 patients of unknown stage (**Supplementary Table 4**). Fifty-one point six percent of NED subjects and 60.4% of CRC patients were males. Across different groups, there was no significant difference for gender distribution, whereas age distribution showed significant difference (**Table 1**).

**Table 1:** Characteristics of subjects enrolled in this study.

Group	Total Number	Gender			Age	
		Male (n [%])	Female (n [%])	*p*	Median (Range)	*p*
**NED**	95	49 [51.6]	46 [48.4]	0.149	48 (22–83)	<0.05
**SP**	49	31 [63.3]	18 [36.7]		55 (24–84)	
**AA**	23	11 [47.8]	12 [52.2]		66 (46–92)	
**CRC**	154	93 [60.4]	61 [39.6]		62.5 (27–89)	

To evaluate the analytical performance of m*CLIP4* test, a series of genomic DNA solutions of different methylation levels were tested in 24 replicates. As shown in **Table 2**, m*CLIP4* test was able to detect as low as 6.75 pg fully methylated genomic DNA (~2 copies of human genome) per PCR reaction. Defined as the concentration at which more than 95% of the replicates generated amplification signals (24), LoD of m*CLIP4* test was approximately 60 pg (~18 copies of human genome) per PCR reaction.

**Table 2:** The analytical performance of m*CLIP4* test.

Fully methylated genomic DNA concentration (pg/reaction)	Detected	Detection rate (%)
Unmethylated genomic DNA	0 out of 24	0.0
6.75	6 out of 24	25.0
12.5	8 out of 24	33.3
25	17 out of 24	70.8
50	22 out of 24	91.7
60	24 out of 24	100.0
70	24 out of 24	100.0

**Figure 2:**
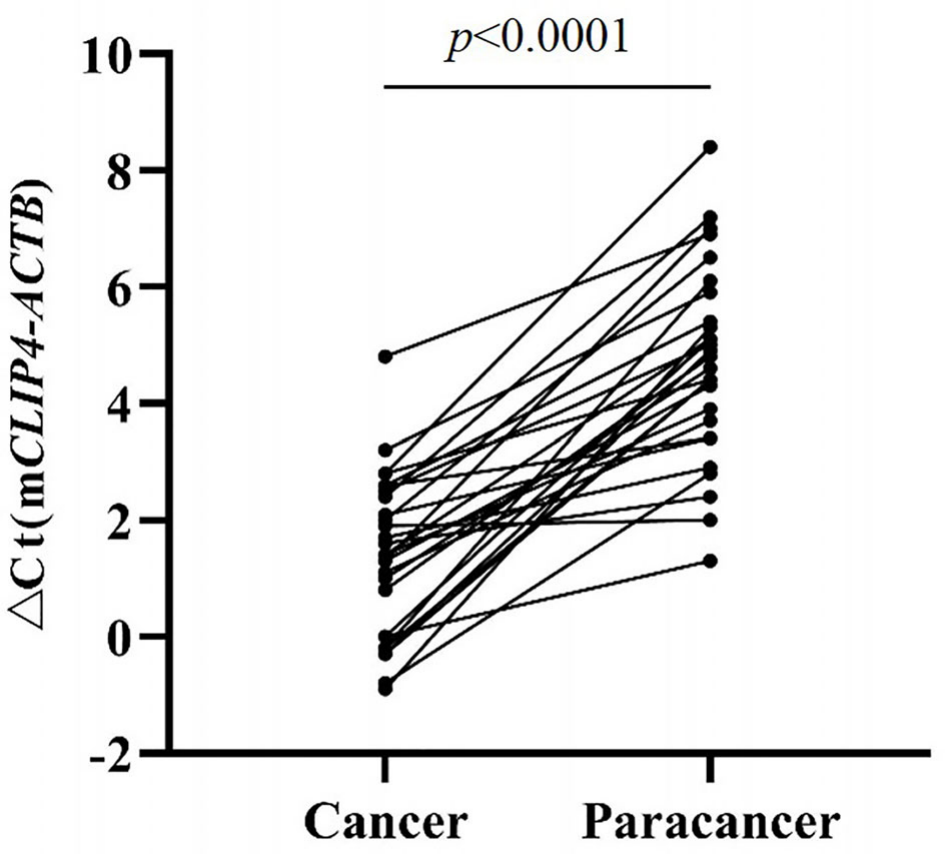
The Methylation levels of *CLIP4* gene in CRC tissues and paired adjacent paracancerous tissues.

**Figure 3:**
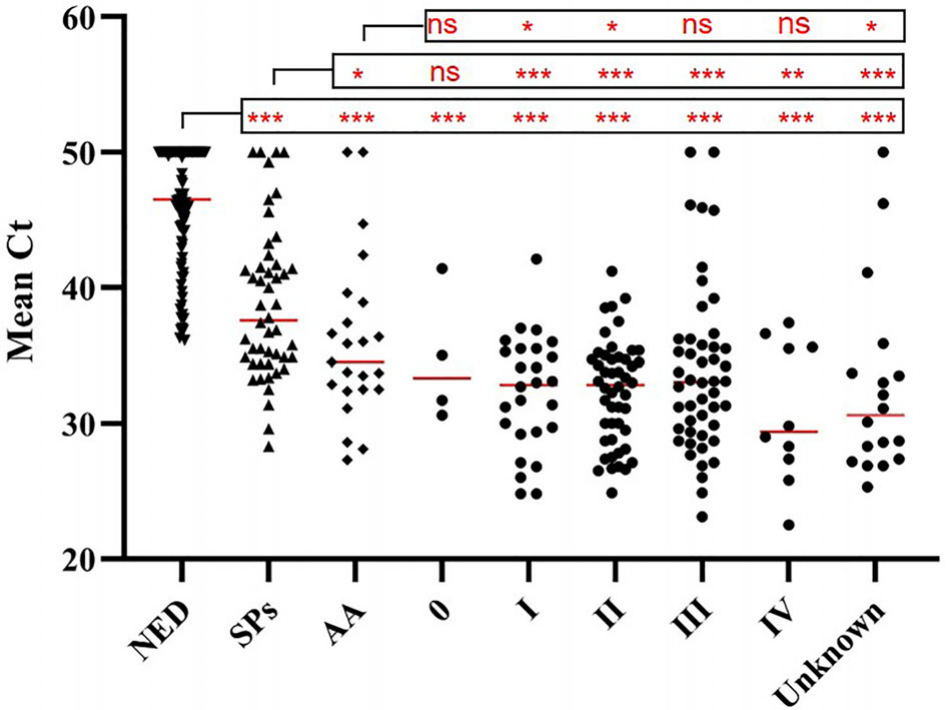
Methylation levels (mean Ct values) of *CLIP4* gene in stool samples from NED subjects, as well as SP, AA and CRC patients of different stages. Horizontal red bars denote the median value of mean Ct values of all samples within the group. ns, not significant. *p < 0.05, **p < 0.01, ***p < 0.0001.

**Figure 4:**
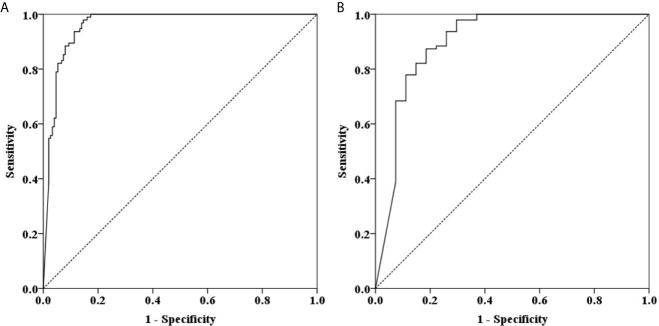
ROC curves for stool m*CLIP4* test for detecting AA **(A)** and CRC **(B)**. AUC values for AA **(A)** and CRC **(B)** were 0.892 (95% CI: 0.795–0.988) and 0.961 (95% CI: 0.938–0.983), respectively.

The results of tissue samples indicated that m*CLIP4* levels in all cancer tissues were higher than those in their paired adjacent paracancerous tissues (*p* < 0.0001, **Figure 2**). Out of 321 subjects diagnosed by colonoscopy and further confirmed for CRC patients by pathological analysis of surgically resected specimens, m*CLIP4* was detected in 11.6% of NED (11/95), 53.1% of SP (26/49), 78.3% of AA (18/23) stool specimens, as well as 75.0% of Stage 0 (3/4), 96.2% of Stage I (25/26), 95.8% of Stage II (46/48), 83.1% of Stage III (40/48), 100% of Stage IV (10/10), and 83.3% CRC samples of unknown stage (15/18) (**Supplementary Table 4**). The overall sensitivities for detecting AA and CRC by m*CLIP4* test were 78.3% (95% CI: 55.8%–91.7%) and 90.3% (95% CI: 84.2%–94.3%), respectively, with a specificity of 88.4% (95% CI: 79.8%–93.8%) (**Table 3**). As shown in **Figure 3**, mean Ct value of each group represented the average methylation level, and a lower Ct value indicated a higher methylation level. Stool m*CLIP4* levels of NED were significantly lower than those of patients with intestinal lesions, including SPs, AAs, and CRCs (*p* < 0.0001). There were no significant differences in m*CLIP4* levels between samples of SP and stage 0 CRC patients (*p* > 0.05), whereas Stage I-IV CRC patient samples showed significantly higher m*CLIP4* levels than those of SP patients. Differences in stool m*CLIP4* levels between AA and stage III-IV CRC patients were not significant, but stage I-II CRC patients showed significantly higher m*CLIP4* levels than those of AA patients. Furthermore, ROC curves of m*CLIP4* test for AA and CRC detection demonstrated its ability to discriminate AA and CRC from controls with AUC values of 0.892 (95% CI: 0.795–0.988) and 0.961 (95% CI: 0.938–0.983) (**Figure 4**).

**Table 3:** The sensitivities and specificities of CRC, AA and SP.

	Sensitivities (95% CI)	Specificity (95% CI)
**CRC**	90.3% (84.2%–94.3%)	88.4% (79.8%–93.8%)*
**AA**	78.3% (55.8%–91.7%)	
**SP**	53.2 (38.4%–67.2%)	

*The specificities for CRC, AA and SP were the same.

Further analysis showed no significant sensitivity difference among different age groups, genders, stages, locations, sides, tumor sizes and differentiation statuses (*p* > 0.05, **Table 4**).

**Table 4:** Sensitivities of stool m*CLIP4* test for detecting CRC for different age groups, genders, stages, tumor locations, sides, tumor sizes and differentiation statuses.

	Total (n)	Positive (n)	Sensitivity (%)	*p*-value
**Age**				
<60	66	58	87.9	0.388
≥60	88	81	92.0	
**Gender**				
Male	93	86	92.5	0.253
Female	61	53	86.9	
**Stage**				
0–II	78	74	94.9	0.078
III–IV	58	50	86.2	
**Location**				
Proximal	61	53	86.9	0.315
Distal	87	80	92.0	
**Colon**				
Left-sided	34	30	88.2	0.877
Right-sided	15	13	86.7	
**Tumor size**				
≤ 4 cm	68	62	91.2	0.867
>4 cm	62	56	90.3	
**Differentiations** ^a^				
Poor	26	23	88.5	0.767
Moderately	78	71	91.0	
Well	19	18	94.7	

^a^p-value was calculated by Pearson chi-square test.

## Discussion

Appropriate screening and surveillance for precancerous lesion and early stage CRC can significantly mitigate CRC mortality, and AA is the preferred target stage. Coverage of guideline-recommended screening in the US has increased to 67%: approximately 61% and 11% of US adults over 50 underwent a colonoscopy or a stool test, respectively, contributing to substantial reduction in morbidity and mortality (4). However, the invasiveness of colonoscopy and limited medical resource per capita have resulted in low compliance rate especially in average-risk and young adults in most countries. In this study, we provided a convenient stool DNA (m*CLIP4*) test as an alternative screening and potential surveillance method.

The cost-effectiveness of FIT and gFOBT, two guideline-compliant screening methods, has been intensively investigated. Previous studies showed the sensitivities of selected commercial FIT and high-sensitivity gFOBT kits for detecting advanced colorectal neoplasia and CRC varied from 7.4% to 57.1%, with relatively high specificities between 96.8% and 98.6% (25, 26). Studies suggested that FIT in consecutive 3 years could play a role in significant cost savings by replacing colonoscopy, with a risk of missing 40% to 70% AAs and 30% to 40% CRCs (27). In comparison, our case-control study showed a much higher sensitivity of 78.3% and 90.3%, respectively, for detecting AA and CRC. As a result, the risk of missed diagnosis would dramatically decrease to approximately 21.7%, 4.7% or 1.0% for AA, and 9.7%, 0.9% or 0.1% for CRC, respectively, if m*CLIP4* tests were performed 1, 2 or 3 times in consecutive years, indicating its potential for early CRC screening.

Numerous studies indicated better performance of DNA methylation markers in stool than those in plasma due to a limited amount of circulating tumor DNA (ctDNA) in plasma and a substantial background of circulating free DNA (cfDNA) from other sources. This was particularly true for the ability to detect precancerous lesions and CRC at early stage. In addition to better performance, stool DNA test offered a feasible solution of at-home cancer screening in populous countries with limited medical resources per capita (28). Furthermore, unlike stool DNA test, blood-based DNA test has not been included in the guideline for routine CRC screening, since its effectiveness has yet to be demonstrated in asymptomatic screening population (29). Comparisons of the performance of the same methylated DNA markers in stool to those in plasma were conducted in several studies. Epi proColon 2.0 assay, the first blood-based m*SEPT9* assay approved by FDA, showed a limited sensitivity of 22% for AA and 68.2% for CRC with a specificity of 78.2% (30). A direct comparison study found significantly higher m*SEPT9* level in stool samples than in plasma. Whereas the performance of both tests in detecting all stage CRC was similar, stool m*SEPT9* test achieved improvement of 35.9% and 7.9% in sensitivity for detecting AA and stage I-II CRC when compared to plasma test (31). Jensen et al. identified three methylation markers, *C9orf50*, *KCNQ5*, and *CLIP4*, and evaluated their performance for CRC detection with plasma samples. Hypermethylation of *CLIP4* by itself showed a 77% sensitivity to discriminate CRC patients from healthy individuals. Multiplex methylation assay of all three markers showed an improved sensitivity of 85% at 99% specificity, and sensitivities of 80%, 85%, 89% and 88% for stage I, stage II, stage III and stage IV CRC, respectively, while lacking data for AA patients (16). Compared to the above plasma multiplex test, our stool m*CLIP4* test demonstrated sensitivities of 78.3% for AA and 90.3% for CRC (96.2% for stage I, 95.8% for stage II, 83.1% for stage III and 100% for stage IV) with a slight compromise in specificity, suggesting it as a promising tool for early CRC screening.

Single-and multi-target stool DNA assays have been developed and evaluated over the past decades. For example, studies on stool-based m*SDC2* tests showed sensitivities ranging from 42.1% to 66.7% for AA and 81.1% to 90.2% for CRC with a 90.2% to 98.0% specificity (24, 32–34). In comparison, m*CLIP4* assay in this study showed better performance for detecting AAs by an increase of 12% to 36% in sensitivity with similar specificity for CRC detection. Furthermore, Wang et al. showed significantly lower sensitivity of 75.6% for detecting stage IV CRC with stool m*SDC2* test, implying the possible preference of m*SDC2* by stages. For m*CLIP4* test, no such preference was observed for stage IV CRC in our limited study. In general, multi-target methylation or methylation-mutation assays were considered capable of reducing false negative rate and improving sensitivity. Cologuard, another FDA-approved molecular diagnostic test for early CRC screening, included assays for 7 *K-RAS* point mutations, aberrant *NDRG4* and *BMP3* methylation with β-actin as a reference gene and a hemoglobin immunoassay. It demonstrated sensitivities of 42.4% for AA and 92.3% for CRC with 86.6% specificity in an extensive study (11). Our previous study also evaluated a combined assay of m*SEPT9* and m*SDC2*, ColoDefence test, resulting in sensitivities of 66.7% for AA and 92.3% for CRC with 93.2% specificity (20). Compared to these two multiplex tests, m*CLIP4* test achieved an even higher sensitivity for AA by an increase of 12% to 36% with a similarly high specificity. In addition, similar to ColoDefence test, only 5 g of stool sample was required for m*CLIP4* test, a single-tube multiplex qPCR assay, leading to reduction of cost and complexity of the procedure.

Stool m*CLIP4* test demonstrated the feasibility for CRC detection, especially in detecting precancerous lesions and early stage CRC in our study. However, this case-control study had several limitations. First, the main purpose of this study was to evaluate the feasibility of stool m*CLIP4* test for CRC detection in a training cohort of a limited number of participants. Further validation and comparison with other existing molecular diagnostic tests in future studies could provide additional support to its potential for CRC screening and prevention. Second, due to limited enrollment, characteristics of the subjects, such as age distribution in different groups, did not reflect the true distribution in a larger population. Although m*CLIP4* level did not correlate with patient age in our study, future studies with larger cohorts may better define this relationship. Third, hypermethylation of *CLIP4* in plasma and/or tissues was also found in other gastrointestinal (GI) cancers, possibly leading to false positives in CRC detection in the presence of other GI cancers. Although degradation of DNA from upper gastrointestinal tract *via* intestine would be expected to significantly reduce the false positive results due to other GI cancers, including stool samples from such patients as control in future studies would help address this concern. Nonetheless, our findings demonstrated that m*CLIP4* stool test may be a promising tool for early CRC detection.

## Conclusions

Stool methylated *CLIP4* test demonstrated high sensitivities in detecting SP, AA and CRC with a high specificity. Its performance on precancerous lesions and early stage CRCs made it a promising biomarker for the early detection of colorectal neoplasms. Small amount of sample needed and single-biomarker assay may also reduce screening cost. Therefore, stool m*CLIP4* test has the potential to become a convenient alternative method for early CRC screening.

## Data Availability Statement

The data sets presented in this study can be found in online repositories. The names of the repository/repositories and accession number(s) can be found in the article/**Supplementary Material**.

## Ethics Statement

This study was performed according to the principles of the Helsinki Declaration and approved by the Institutional Review Board of the Affiliated Hospital of Xuzhou Medical University (Ethics Committee reference number: XYKY2020-KL156-01). All participants have acknowledged and signed the informed consent. The patients/participants provided their written informed consent to participate in this study.

## Author Contributions

YC, GZ, YPC, SX, and YM performed the statistical analyses and drafted the manuscript. YC, YPC, ZC, XL, MY, JY, XW, ZL, MZ, and SF participated in sample collection and data analysis. GZ, SX, MZ, and SF conceived of the study and participated in the design and coordination of the study. All authors contributed to the article and approved the submitted version.

## Funding

The work was supported by the grants from the Suzhou Technology Entrepreneur Angel Project (grant CYTS2018051), Key Technologies R & D Program for Social Development of Jiangsu Province (grant BE2019688), Kunshan Leading Talent Project (grant 00311), Suzhou Innovation and Entrepreneurship Leading Talent Program (grant ZXL2020046), and Key Technologies R & D Program for Social Development of Xuzhou (grant KC17184).

## Conflict of Interest

GZ and SX are employees of Suzhou VersaBio Technologies Co. Ltd. SX is a shareholder of Suzhou VersaBio Technologies Co. Ltd.

The remaining authors declare that the research was conducted in the absence of any commercial or financial relationships that could be construed as a potential conflict of interest.

## Abbreviations

CRC, colorectal cancer; AA, advanced adenomas; SP, small polyps; AUC, area under curve; FOBT, fecal occult blood test; FIT, fecal immunochemical test; sDNA, stool DNA; FDA, Food and Drug Administration; *CLIP*, CAP-Gly domain containing linker protein; m*CLIP4*, methylated *CLIP4*; NED, no evidence of disease; LoD, Limit of detection; ROC, receiver operating characteristic; ctDNA, circulating tumor DNA; cfDNA, circulating free DNA.
