# Supplementary material for: Feasibility of Methylated CLIP4 in Stool for Early Detection of Colorectal Cancer: A Training Study in Chinese Population
Source: Front Oncol. 2021 Apr 22;11:647066. doi: 10.3389/fonc.2021.647066 (PMC8100593; doi:10.3389/fonc.2021.647066)
Supplement: Supplementary file 1 [file Presentation_1.pptx]

## Slide 1
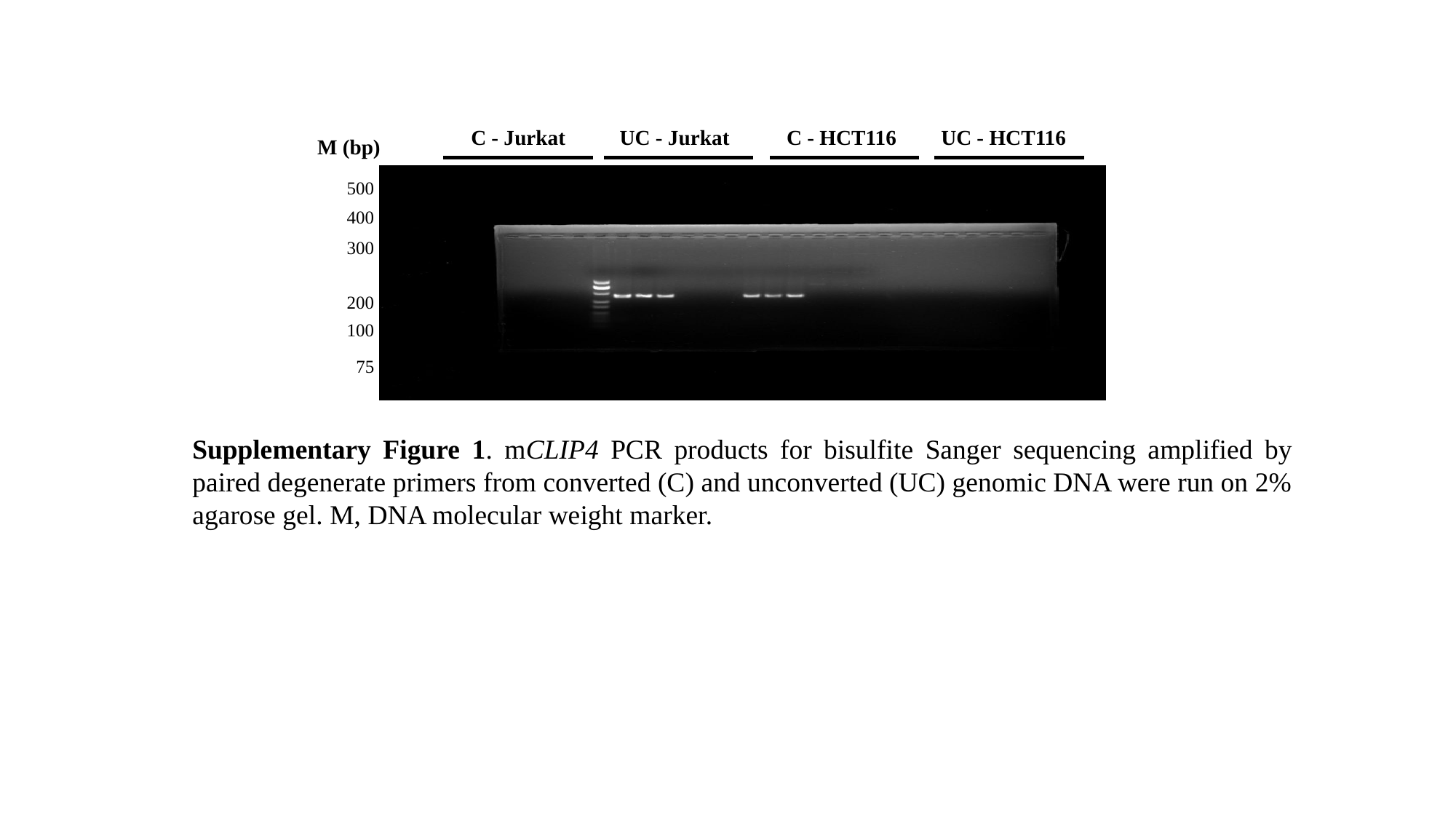

C - Jurkat
UC - Jurkat
C - HCT116
UC - HCT116
M (bp)
500
400
300
200
100
75
Supplementary Figure 1. mCLIP4 PCR products for bisulfite Sanger sequencing amplified by paired degenerate primers from converted (C) and unconverted (UC) genomic DNA were run on 2% agarose gel. M, DNA molecular weight marker.

## Slide 2
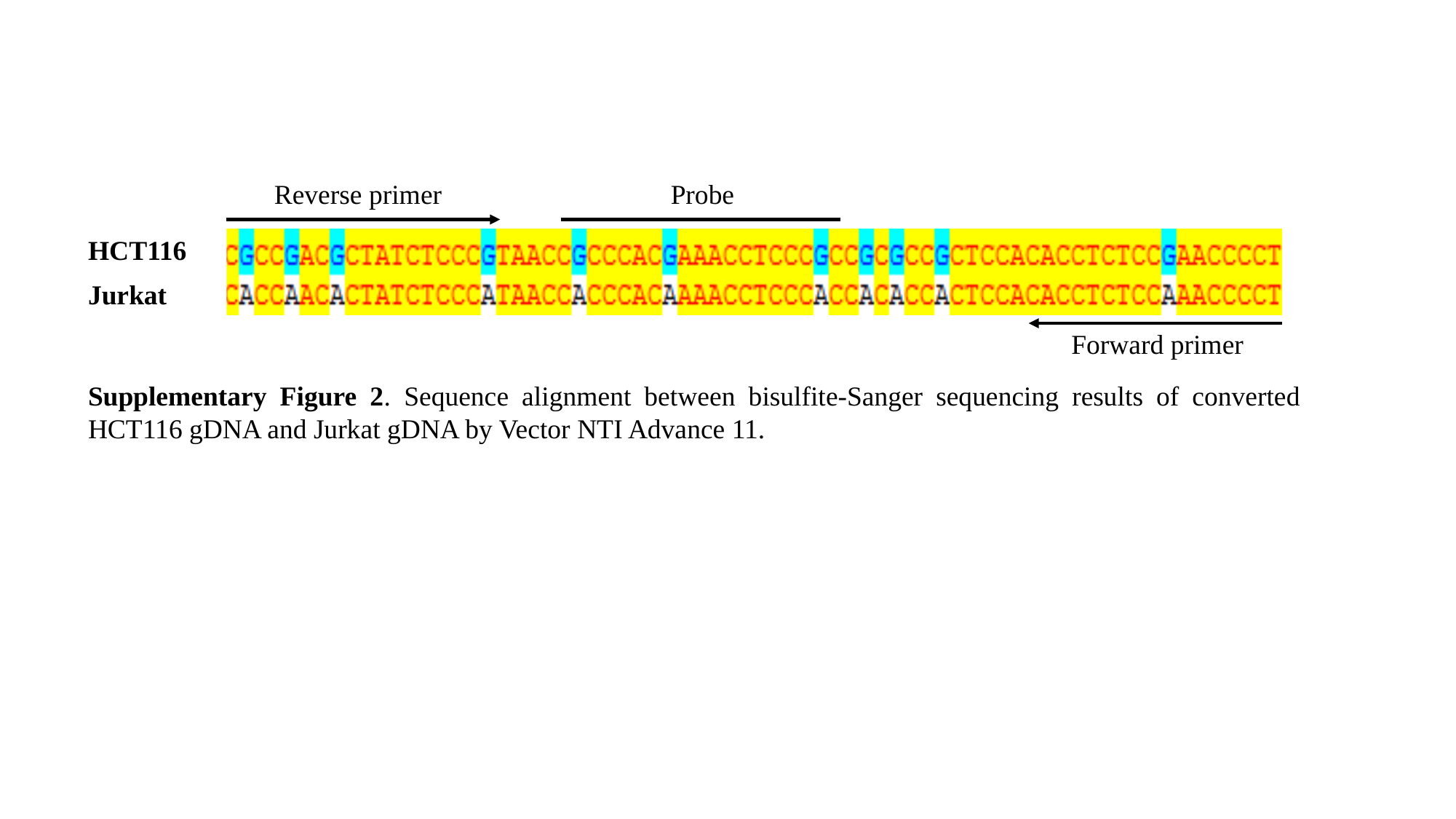

Reverse primer
Probe
HCT116
Jurkat
Forward primer
Supplementary Figure 2. Sequence alignment between bisulfite-Sanger sequencing results of converted HCT116 gDNA and Jurkat gDNA by Vector NTI Advance 11.
